# Association of Pregnancy-Specific Alcohol Policies With Infant Morbidities and Maltreatment

**DOI:** 10.1001/jamanetworkopen.2023.27138

**Published:** 2023-08-03

**Authors:** Sarah C. M. Roberts, Alex Schulte, Claudia Zaugg, Douglas L. Leslie, Tammy E. Corr, Guodong Liu

**Affiliations:** 1 Department of Obstetrics, Gynecology, and Reproductive Sciences, University of California, San Francisco, Oakland; 2 Department of Public Health Sciences, Penn State College of Medicine, Hershey, Pennsylvania; 3 Department of Pediatrics, Penn State College of Medicine, Hershey, Pennsylvania

## Abstract

**Importance:**

Research has found associations of pregnancy-specific alcohol policies with increased low birth weight and preterm birth, but associations with other infant outcomes are unknown.

**Objective:**

To examine the associations of pregnancy-specific alcohol policies with infant morbidities and maltreatment.

**Design, Setting, and Participants:**

This retrospective cohort study used outcome data from Merative MarketScan, a national database of private insurance claims. The study cohort included individuals aged 25 to 50 years who gave birth to a singleton between 2006 and 2019 in the US, had been enrolled 1 year before and 1 year after delivery, and could be matched with an infant. Data were analyzed from August 2021 to April 2023.

**Exposures:**

Nine state-level pregnancy-specific alcohol policies obtained from the National Institute on Alcohol Abuse and Alcoholism’s Alcohol Policy Information System.

**Main Outcomes and Measures:**

The primary outcomes were 1 or more infant injuries associated with maltreatment and infant morbidities associated with maternal alcohol consumption within the first year. Logistic regression, adjusting for individual-level and state-level controls, and fixed effects for state, year, state-specific time trends, and SEs clustered by state were used.

**Results:**

A total of 1 432 979 birthing person–infant pairs were included (mean [SD] age of birthing people, 32.2 [4.2] years); 30 157 infants (2.1%) had injuries associated with maltreatment, and 44 461 (3.1%) infants had morbidities associated with alcohol use during pregnancy. The policies of Reporting Requirements for Assessment/Treatment (adjusted odds ratio [aOR], 1.28; 95% CI, 1.08-1.52) and Mandatory Warning Signs (aOR, 1.18; 95% CI, 1.10-1.27) were associated with increased odds of infant injuries but not morbidities. Priority Treatment for Pregnant Women Only was associated with decreased odds of infant injuries (aOR, 0.83; 95% CI, 0.76-0.90) but not infant morbidities. Civil Commitment was associated with increased odds of infant injuries (aOR, 1.26; 95% CI, 1.08-1.48) but decreased odds of infant morbidities (aOR, 0.57; 95% CI, 0.53-0.62). Priority Treatment for Pregnant Women and Women With Children was associated with increased odds of both infant injuries (aOR, 1.12; 95% CI, 1.00-1.25) and infant morbidities (aOR, 1.08; 95% CI, 1.03-1.13). Reporting Requirements for Child Protective Services, Reporting Requirements for Data, Child Abuse/Neglect, and Limits on Criminal Prosecution were not associated with infant injuries or morbidities.

**Conclusions and Relevance:**

In this cohort study, most pregnancy-specific alcohol policies were not associated with decreased odds of infant injuries or morbidities. Policy makers should not assume that pregnancy-specific alcohol policies improve infant health.

## Key Points

**Question:**

What is the association of state-level pregnancy-specific alcohol policies with infant morbidities and maltreatment?

**Findings:**

In this population-level cohort study of 1 432 979 birthing person–infant pairs in the US, most state-level pregnancy-specific alcohol policies were not associated with decreased odds of infant maltreatment or morbidities. The few policies that were associated with decreased odds of maltreatment or morbidities were associated with an increase in the odds of another adverse outcome.

**Meaning:**

These findings suggest that state-level pregnancy-specific alcohol policies do not appear to be effective at reducing harms to infants.

## Teaser

This cohort study examines the associations of pregnancy-specific alcohol policies with infant morbidities and maltreatment.

## Introduction

The adverse outcomes of pregnant people’s alcohol consumption remain a substantial public health concern in the US and globally.^1,2,3^ Despite most US states having multiple policies in effect that attempt to reduce alcohol use during pregnancy and related harms,^4^ rates of use during pregnancy and related harms have remained steady.^5,6,7,8,9,10^

Previous research^11^ has examined associations of pregnancy-specific alcohol policies with alcohol use during pregnancy and birth outcomes and found that pregnancy-specific alcohol policies are largely not associated with alcohol use during pregnancy. Research has also found that multiple pregnancy-specific alcohol policies—including Mandatory Warning Signs, Child Abuse/Neglect, Limits on Criminal Prosecution, and Priority Treatment for Pregnant Women—are associated with increased low birth weight and preterm birth and decreased prenatal care (see eTable 1 in Supplement 1 for definitions of the pregnancy-specific alcohol policies referenced in this article).^12,13^ This research suggests that pregnancy-specific alcohol policies are mostly ineffective and possibly harmful to general public health outcomes.

Previous literature on associations of pregnancy-specific alcohol policies with alcohol use is constrained by small numbers of pregnant people in national surveys that measure alcohol consumption and reliance on self-reported measures of alcohol consumption,^11^ in which willingness to report use could be influenced by the policy environment itself. This previous research also reports on outcomes (eg, low birth weight and preterm birth)^13^ that are not specific foci of policies and may include neither the specific birth defects associated with alcohol use during pregnancy nor child maltreatment, which pregnancy-specific alcohol policies target.

Research to understand whether findings from extant literature extend to other outcomes, including those specifically targeted by pregnancy-specific alcohol policies, is needed. The present study used a private insurance claims database to examine the associations of pregnancy-specific alcohol policies with infant injuries associated with maltreatment, infant morbidities, maternal morbidities, and infant health care utilization.

## Methods

### Study Design

This retrospective cohort study examined the associations of state-level pregnancy-specific alcohol policies with infant and maternal outcomes and infant health care utilization. The University of California, San Francisco, institutional review board considered this deidentified data study exempt, and the Penn State Institutional Review Board considered this study not human participants research; thus, informed consent was not needed in accordance with 45 CFR §46. We followed Strengthening the Reporting of Observational Studies in Epidemiology (STROBE) reporting guideline.

### Data Sources

This study used policy data from the National Institute on Alcohol Abuse and Alcoholism’s Alcohol Policy Information System^4^ and outcome data from the Merative MarketScan Commercial Claims and Encounters database, a commercially available health insurance claims database. MarketScan contains claims for a sample of privately insured people in all 50 US states and the District of Columbia, including demographic characteristics, health care utilization, dates of service, diagnosis codes, procedure codes, and facility type. Data represent claims that have been adjudicated for payment and are obtained directly from a convenience sample of health plans and large employers that agree to participate in MarketScan.

### Study Population and Exposures

The study population included all reproductive-aged female beneficiaries in the database aged 12 to 50 years who gave birth to a singleton between 2006 and 2019, had been continuously enrolled 1 year before and 1 year after delivery, could be matched under the same household with an infant who had at least 1 claim within the first month after delivery and was continuously enrolled for 1 year after birth, and resided in a US state or Washington, DC. Primary independent variables include dichotomous pregnancy-specific alcohol policies as specified by the National Institute on Alcohol Abuse and Alcoholism^4^: Reporting Requirements for Child Protective Services (CPS), Reporting Requirements for Assessment/Treatment, Reporting Requirements for Data, Mandatory Warning Signs, Child Abuse/Neglect, Limits on Criminal Prosecution, Civil Commitment, Priority Treatment for Pregnant Women Only, and Priority Treatment for Pregnant Women and Women With Children (see eTable 1 in Supplement 1 for definitions of the pregnancy-specific alcohol policies).

### Outcomes

Primary outcomes include infant injuries associated with maltreatment and infant morbidities associated with alcohol use during pregnancy, each dichotomous. Infant injuries include having 1 or more *International Classification of Diseases, Ninth Revision (ICD-9)* and *International Statistical Classification of Diseases and Related Health Problems, Tenth Revision (ICD-10)* codes that previous research^14^ has found to have positive predictive value of greater than 50% for child maltreatment (eTable 2 in Supplement 1). Infant morbidities include having 1 or more *ICD-9* and *ICD-10* codes for infant morbidities that previous research^15^ has identified as being related to alcohol use during pregnancy (eTable 3 in Supplement 1).

Secondary outcomes include severe maternal morbidities, defined as having 1 or more *ICD-9* and *ICD-10* codes for a de novo severe maternal morbidity within 6 weeks after discharge from the delivery hospital (eTable 4 in Supplement 1).^16,17^ They also include 3 infant health care utilization outcomes: inadequate well-child visits (<4 well-child visits before the first birthday, informed by American Academy of Pediatrics^18^ and California Department of Health Care Services^19^ guidelines and data distribution) and 2 or more emergency department (ED) visits and 2 or more inpatient admissions (each dichotomous of ≥2 of this type of health care utilization during infant’s first year).

Individual-level controls include the birthing person’s age (categorized as 25-29, 30-34, 35-39, 40-44, and ≥45 years) and health status (Elixhauser Comorbidity Index,^20^ categorized as 0, 1, 2, and ≥3 comorbidities). State-level controls include unemployment, poverty, per capita alcohol consumption, and per capita tobacco consumption.^21,22,23,24,25^ For sensitivity analyses examining pregnancy-specific drug policies, we also included legal recreational cannabis.^4^ Pregnancy-specific alcohol policy data were merged with individual-level outcome data according to the estimated month and year the person became pregnant, accounting for preterm births.

### Statistical Analysis

Data were analyzed from August 2021 to April 2023. Multivariable logistic regression models examined associations between policies and individual-level outcomes. Models included all policy indicators simultaneously; adjusted for individual-level controls and, when Wald tests indicated state controls improved model fit, state-level controls; included fixed effects for state and year, as well as state-specific time trends; and accounted for clustering of SEs according to residence state. Unadjusted findings from models examining each policy in separate models are in eTable 5 in Supplement 1. The analysis assumes that missing data for the study cohort are rare because these are adjudicated billing claims used to determine payments to clinicians, hospitals, and pharmacies. Analyses were performed in Stata statistical software version 16.1 (StataCorp) and used a 2-sided statistical significance level of *P* < .05.

A series of sensitivity analyses were conducted. First, analyses examined whether findings were sensitive to changing the definition of when a policy was considered to be in effect for a given birthing person–infant pair from at conception to at birth. Second, analyses examined whether findings were sensitive to assumptions, including inclusion vs exclusion of birthing people younger than 25 years (because of the limited ability to match younger birthing people with infants), subsequent pregnancies, Civil Commitment and Limits on Criminal Prosecution (rarer policies that often coexist with other policies), and all state controls. Finally, pregnancy-specific drug policies were considered. Per a priori study plans, pregnancy-specific alcohol policies were established as main policies of interest. However, most state policies covering alcohol use during pregnancy also cover drugs.^26^ Because of this overlap, separately including pregnancy-specific alcohol and pregnancy-specific drug policies is infeasible. Instead, sensitivity analyses examined (1) pregnancy-specific drug and (2) pregnancy-specific alcohol and/or drug policies.

## Results

**Figure 1.**
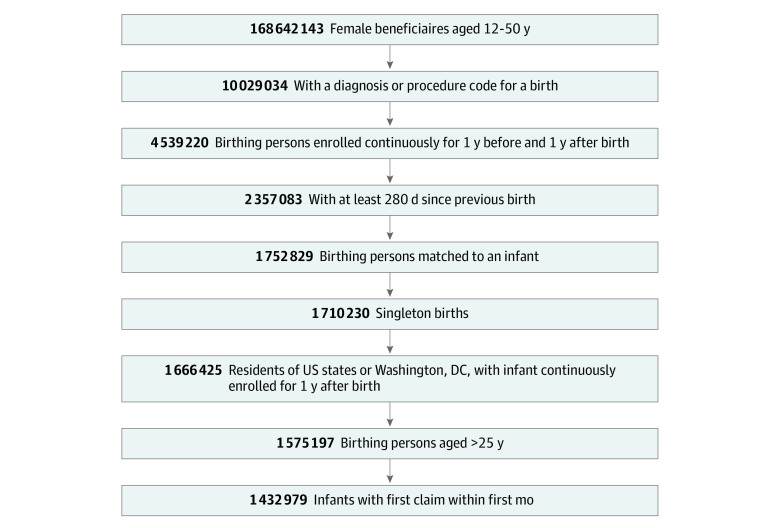
Flow Diagram for Cohort Extraction

We identified 1 666 425 singleton birth and birthing person pairs that met inclusion criteria. We then excluded all births among people younger than age 25 years (91 228 births), because more than 70% were not matched with an infant. We excluded births where the infant did not have a claim within the first month after birth (142 218 births), because data on key study outcomes—particularly infant morbidities—appeared to be missing for infants without a claim within the first month. The final study cohort included a total of 1 432 979 birthing person-infant dyads (Figure 1).

**Figure 2.**
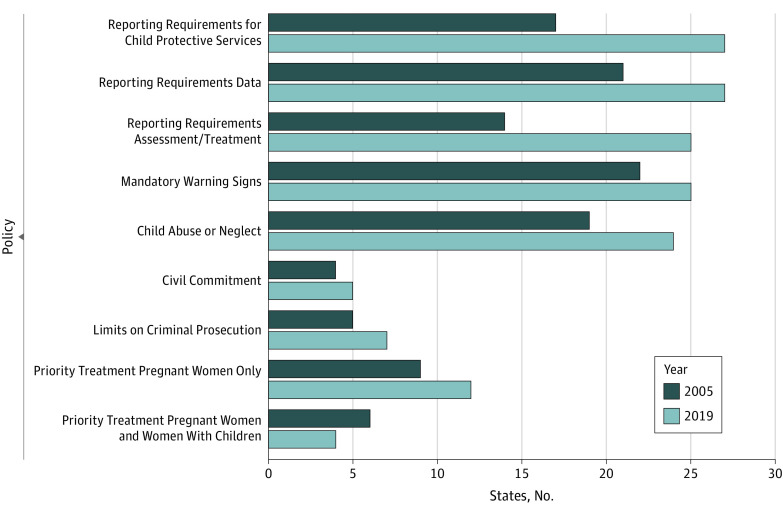
Number of States With Pregnancy-Specific Alcohol Policies 2005 and 2019

In the study cohort, mean (SD) age of birthing people was 32.2 (4.2) years. Most (972 968 individuals [67.9%]) had no Elixhauser comorbidities whereas 37 630 (2.6%) had 3 or more comorbidities (Table 1). The number of states with each pregnancy-specific policy increased between 2005 and 2019, except Priority Treatment for Pregnant Women and Women With Children (Figure 2). More than 40% of birthing people were exposed to Reporting Requirements for CPS, Reporting Requirements for Data, or Reporting Requirements for Assessment/Treatment; Child Abuse/Neglect; or Mandatory Warning Signs. Between 10% and 25% were exposed to Priority Treatment, with more exposed to Priority Treatment for Pregnant Women Only than to Pregnant Women and Women With Children. Fewer than 10% were exposed to Civil Commitment or Limits on Criminal Prosecution (Table 1 and eTable 6 in Supplement 1).

**Table 1.** Sample Description of Birthing Individuals and Their Infants

Characteristic	Beneficiaries, No. (%) (N = 1 432 979)
Age, mean (SD), y	32.2 (4.2)
Health status, No. of Elixhauser comorbidities	
0	972 968 (67.9)
1	326 908 (22.8)
2	95 473 (6.7)
≥3	37 630 (2.6)
Policy exposure^a^	
Reporting Requirements for Child Protective Services	603 578 (42.1)
Reporting Requirements Data	848 615 (59.2)
Reporting Requirements Assessment/Treatment	638 965 (44.6)
Mandatory Warning Signs	773 430 (54.0)
Child Abuse or Neglect	652 769 (45.6)
Civil Commitment	61 633 (4.3)
Limits on Criminal Prosecution	135 968 (9.5)
Priority Treatment Pregnant Women Only	362 879 (25.3)
Priority Treatment Pregnant Women and Women With Children	162 584 (11.4)
Infant injuries consistent with maltreatment	30 157 (2.1)
Infant morbidities associated with alcohol use during pregnancy	44 461 (3.1)
Severe maternal morbidities	47 582 (3.3)
Inadequate well-child visits	148 170 (10.3)
≥2 Emergency department visits	92 586 (6.5)
≥2 Inpatient admissions	32 345 (2.3)

^a^ Policies are defined by the National Institute on Alcohol Abuse and Alcoholism’s Alcohol Policy Information System (eTable 1 in Supplement 1).

Among the infants, 30 157 (2.1%) had an injury associated with maltreatment and 44 461 (3.1%) had a morbidity associated with alcohol use during pregnancy; 47 582 birthing people (3.3%) experienced a severe maternal morbidity. Regarding health care utilization, 148 170 infants (10.3%) had fewer than 4 well-child visits, 92 586 (6.5%) had 2 or more ED visits, and 32 345 (2.3%) had 2 or more inpatient hospitalizations.

### Injuries and Morbidities

In adjusted models, Reporting Requirements for Assessment/Treatment was associated with increased odds of infant injuries (adjusted odds ratio [aOR], 1.28; 95% CI, 1.08-1.52), but not infant or maternal morbidities (Table 2 and eTable 7 in Supplement 1). Mandatory Warning Signs was associated with increased odds of infant injuries (aOR, 1.18; 95% CI, 1.10-1.27) and maternal morbidities (aOR, 1.87; 95% CI, 1.72-2.03), but not infant morbidities. Civil Commitment was associated with increased odds of infant injuries (aOR, 1.26; 95% CI, 1.08-1.48), but decreased odds of infant morbidities (aOR, 0.57; 95% CI, 0.53-0.62), and was not associated with maternal morbidities. Priority Treatment for Pregnant Women Only was associated with decreased odds of infant injuries (aOR, 0.83; 95% CI, 0.76-0.90), but not infant or maternal morbidities. Priority Treatment for Pregnant Women and Women With Children was associated with increased odds of infant injuries (aOR, 1.12; 95% CI, 1.00-1.25) and infant morbidities (aOR, 1.08; 95% CI, 1.03-1.13), but decreased odds of severe maternal morbidities (aOR, 0.83; 95% CI, 0.70-0.97). Reporting Requirements for CPS, Reporting Requirements for Data, Child Abuse/Neglect, and Limits on Criminal Prosecution were not associated with infant injuries or with infant or maternal morbidities.

**Table 2.** Associations of Pregnancy-Specific Alcohol Policies With Infant Injuries, Infant Morbidities, and Severe Maternal Morbidities

Policy^b^	aOR (95% CI)^a^		
	Infant injuries associated with maltreatment	Infant morbidities associated with alcohol use during pregnancy	Severe maternal morbidities
Reporting Requirements for Child Protective Services	1.00 (0.76-1.31)	0.96 (0.79-1.18)	1.19 (0.73-1.94)
Reporting Requirements Data	0.94 (0.81-1.09)	1.05 (0.96-1.15)	0.95 (0.56-1.60)
Reporting Requirements Assessment/Treatment	1.28 (1.08-1.52)	1.04 (0.94-1.16)	1.35 (0.92-2.00)
Mandatory Warning Signs	1.18 (1.10-1.27)	1.01 (0.93-1.11)	1.87 (1.72-2.03)
Child Abuse or Neglect	0.82 (0.63-1.07)	1.02 (0.89-1.17)	0.69 (0.30-1.62)
Civil Commitment	1.26 (1.08-1.48)	0.57 (0.53-0.62)	1.10 (0.87-1.38)
Limits on Criminal Prosecution	1.12 (0.98-1.29)	1.10 (0.96-1.26)	1.05 (0.75-1.48)
Priority Treatment Pregnant Women Only	0.83 (0.76-0.90)	0.97 (0.89-1.05)	1.07 (0.99-1.16)
Priority Treatment Pregnant Women and Women With Children	1.12 (1.00-1.25)	1.08 (1.03-1.13)	0.83 (0.70-0.97)

Abbreviation: aOR, adjusted odds ratio.

^a^ Models include individual-level controls (age and health status), state and year fixed effects, and state-specific time trends and account for clustering by state. The infant injuries model also controls for state-level unemployment.

^b^ Policies are defined by the National Institute on Alcohol Abuse and Alcoholism’s Alcohol Policy Information System (eTable 1 in Supplement 1).

The findings were somewhat sensitive to models examining policies in effect when people gave birth. Specifically, associations of Mandatory Warning Signs and Civil Commitment with infant injuries and of Priority Treatment for Pregnant Women and Women With Children with maternal morbidities were no longer statistically significant (eTable 8 in Supplement 1). Two reporting policies (Reporting Requirements for CPS and Reporting Requirements for Data) that had not been associated with outcomes in main models were associated with increased infant morbidities and severe maternal morbidities. Infant morbidity findings were generally robust to policy timing, except Reporting Requirements for Data (with increased infant morbidities) and Priority Treatment for Pregnant Women Only (with decreased infant morbidities), both of which were statistically significant only when examining policies in effect when people gave birth. Findings were robust for all policies when testing modeling assumptions (data not shown), with the exception of Priority Treatment for Pregnant Women and Women With Children, which was no longer statistically significant in 1 or more sensitivity analyses.

Most findings did not vary when we examined drug or drug and/or alcohol policies. When findings varied, it was typically loss of statistical significance in drug-focused policy models. Other differences included Civil Commitment, for which severe maternal morbidities were newly associated with increased morbidities in the alcohol and/or drug policy model. The only change in direction was Mandatory Warning Signs, which was associated with decreased infant injuries in drug policy models but was associated with increased infant injuries in alcohol policy and alcohol and/or drug policy models (eTable 9 and eTable 10 in Supplement 1).

### Health Care Utilization

In adjusted models, Reporting Requirements for Assessment/Treatment (aOR, 0.81; 95% CI, 0.69-0.95) and Priority Treatment for Pregnant Women and Women With Children (aOR, 0.87; 95% CI, 0.83-0.92) were associated with decreased odds of 2 or more inpatient admissions but not other utilization (Table 3 and eTable 11 in Supplement 1). Mandatory Warning Signs was associated with increased odds of inadequate well-child visits (aOR, 1.12; 95% CI, 1.01-1.24) and decreased odds of 2 or more inpatient admissions (aOR, 0.84; 95% CI, 0.79-0.90), but was not associated with 2 or more ED visits. Civil Commitment was associated with increased odds of 2 or more ED visits (aOR, 1.31; 95% CI, 1.21-1.43) but not other utilization. Limits on Criminal Prosecution was associated with decreased odds of 2 or more ED visits (aOR, 0.82; 95% CI, 0.77-0.87) and 2 or more inpatient admissions (aOR, 0.88; 95% CI, 0.82-0.94). Priority Treatment for Pregnant Women Only was associated with increased odds of inadequate well-child visits (aOR, 1.13; 95% CI, 1.03-1.23) but not other utilization. Reporting Requirements for CPS, Reporting Requirements for Data, and Child Abuse/Neglect were not associated with any utilization outcomes.

**Table 3.** Associations of Pregnancy-Specific Alcohol Policies With Infant Health Care Utilization

Policy^b^	aOR (95% CI)^a^		
	Inadequate well-child visits	≥2 Emergency department visits	≥2 Inpatient admissions
Reporting Requirements for Child Protective Services	0.83 (0.66-1.03)	1.09 (0.99-1.20)	1.24 (0.92-1.68)
Reporting Requirements Data	0.94 (0.80-1.11)	1.09 (0.98-1.22)	1.01 (0.89-1.15)
Reporting Requirements Assessment/Treatment	0.97 (0.83-1.14)	0.99 (0.92-1.07)	0.81 (0.69-0.95)
Mandatory Warning Signs	1.12 (1.01-1.24)	1.03 (0.96-1.11)	0.84 (0.79-0.90)
Child Abuse or Neglect	1.12 (0.86-1.45)	1.04 (0.88-1.23)	0.94 (0.73-1.20)
Civil Commitment	0.98 (0.80-1.21)	1.31 (1.21-1.43)	1.12 (0.98-1.27)
Limits on Criminal Prosecution	0.91 (0.79-1.06)	0.82 (0.77-0.87)	0.88 (0.82-0.94)
Priority Treatment Pregnant Women Only	1.13 (1.03-1.23)	0.99 (0.95-1.03)	0.91 (0.88-0.95)
Priority Treatment Pregnant Women and Women With Children	1.06 (0.90-1.24)	0.99 (0.93-1.04)	0.87 (0.83-0.92)

Abbreviation: aOR, adjusted odds ratio.

^a^ Models include individual-level controls (age, health status), state and year fixed effects, and state-specific time trends and account for clustering by state. The emergency department visits model also controls for state-level unemployment and poverty; the inpatient admissions model also controls for state-level per capita tobacco consumption.

^b^ Policies are defined by the National Institute on Alcohol Abuse and Alcoholism’s Alcohol Policy Information System (eTable 1 in Supplement 1).

The findings were sensitive to models examining policies in effect when people gave birth. Findings for 4 policies for inadequate well-child visits, 3 policies for 2 or more ED visits, and 7 policies for 2 or more inpatient admissions differed when timing of policies was varied. For most, these were variations in statistical significance or effect magnitude; for Mandatory Warning Signs, however, the direction for 2 or more ED visits changed when examining policies in effect when people gave birth (eTable 8 in Supplement 1).

When we examined the modeling assumptions, all findings for inadequate well-child visits were robust, but findings for 2 or more ED visits and 2 or more inpatient admissions were not, although these changes were mostly statistical significance rather than effect magnitude or direction. The only exception was Civil Commitment, for which the association was in the opposite direction in the model without Limits on Criminal Prosecution (data not shown).

With the exception of 2 or more ED visits, utilization findings were somewhat sensitive to examinations of drug or alcohol and/or drug policies. Findings for 2 or more inpatient admissions were more sensitive to drug vs alcohol focus of policies. Most changes were statistical significance and not effect direction or magnitude. The only exception was for Mandatory Warning Signs, for which findings for drug-focused Mandatory Warning Signs was in the opposite direction (eTable 9 and eTable 10 in Supplement 1).

## Discussion

In this retrospective cohort study, 4 of the 9 state-level pregnancy-specific alcohol policies, including Reporting Requirements for CPS, Reporting Requirements for Data, Child Abuse/Neglect, and Limits on Criminal Prosecution, were not associated with infant maltreatment or infant or maternal morbidities. Two policies, Reporting Requirements for Assessment/Treatment and Mandatory Warning Signs, were associated with increased infant maltreatment and/or infant or maternal morbidities without being offset by a decrease in another outcome. To the extent any pregnancy-specific alcohol policy was associated with improvements in infant or maternal outcomes or health care utilization, that policy was associated with increased odds of another outcome. Although some individual associations of policies with outcomes were not robust across sensitivity analyses, sensitivity analyses did not change overall interpretation of findings.

Our findings that state-level pregnancy-specific alcohol policies are generally not associated with improved health outcomes and, in some cases, are associated with increased adverse outcomes are consistent with previous literature^11,13^ examining associations of these policies with alcohol use during pregnancy, birth outcomes, and prenatal care utilization. These findings are also consistent with the slightly larger body of literature examining pregnancy-specific drug policies, which also tends to find few infant health or health care utilization benefits associated with pregnancy-specific drug policies.^27,28^ Combined with previous research, these results provide further evidence that adopting more of the extant type of pregnancy-specific alcohol policies should be paused and that repeal, or at least revision and improvement, of some policies is warranted.

The fact that most findings did not differ between pregnancy-specific drug and pregnancy-specific alcohol policies likely reflects that pregnancy-specific substance-use policies typically cover both alcohol and drugs^26^ and that policy makers passing them may not know whether they cover alcohol, drugs, or both.^29^ The findings appear to apply to both pregnancy-specific alcohol and pregnancy-specific drug policies, noting that sensitivity analyses were not conducted for drug-focused policies and that the infant morbidity outcome related to alcohol consumption during pregnancy. The only exception is Mandatory Warning Signs, for which findings substantively differed between alcohol-focused vs drug-focused policies and which historically have existed for alcohol, but not drugs.^26^

Although it was not the main focus, the prevalence of infant injuries, infant morbidities, and severe maternal morbidities (2%-3%) provides additional evidence that infants and birthing people face health burdens. Pregnancy-specific alcohol policies do not appear to reduce these burdens for infants or birthing people and may exacerbate the problems. Alternative policy approaches that improve infant and maternal health and well-being are urgently needed.

### Strengths and Limitations

This study has strengths. First, this study uses policy data rigorously coded for social science research. Second, outcomes are based on claims data, which is one of the few options for assessing study outcomes. Although an ideal study would examine fetal alcohol spectrum disorder directly, fetal alcohol spectrum disorder surveillance systems vary considerably across states and are not in place in all states; there are also considerable concerns with variations among and underreporting in these systems.^30,31^ Although national databases tracking child maltreatment exist,^32^ these databases track reports to CPS and outcomes of these reports rather than maltreatment. The reports are also likely influenced by the CPS reporting policies we propose to study^33^ and, thus, may measure policy compliance, rather than maltreatment. Third, this study examined infant and maternal outcomes together, rather than separately, which places focus on the family unit, a more relevant way to assess health and well-being of caregiver and child.

There are also limitations to this study. First, infant outcome measurement is imprecise. For infant injuries consistent with maltreatment, we used *ICD-9* and *ICD-10* codes with a positive predictive value of 50%, rather than 75%, with maltreatment. This meant we had sufficient sample for analyses but we made assumptions about injury causes. Second, we were unable to restrict analyses to people with *ICD-9* and *ICD-10* codes for alcohol-related diagnoses or medications because of the rarity of alcohol-related codes in the data. Thus, examined injuries and morbidities cannot be assumed to be due to alcohol consumption during pregnancy. Third, these claims data are from people with employer-sponsored insurance; the findings may not generalize to people with other insurance. Fourth, we are missing individual controls, such as economic status. Given that all data were from people covered by employer-sponsored insurance, there may be less variation in economic status. None of the missing individual-level control variables, however, would be expected to confound associations of state-level policies with outcomes. Fifth, although the analysis cohort can be assumed to have nearly complete data, given that these are adjudicated claims, having to exclude people from the cohort may have influenced the findings. Sixth, the main analyses excluded birthing people younger than 25 years because so few of them were matched with an infant. This likely reflects health insurance patterns for young people, many of whom are still covered under a parent’s insurance and, thus, are unable to place their infant on the same insurance. Sensitivity analyses examining whether inclusion of the young birthing people we could match with an infant did not substantively affect the findings.

## Conclusions

In this retrospective cohort study, most pregnancy-specific alcohol policies were not associated with decreased infant injuries or morbidities or maternal morbidities, and were sometimes associated with increased odds of at least 1 adverse infant or maternal outcome. Policy makers should not assume that pregnancy-specific alcohol policies improve infant or maternal health. Policy approaches more likely to improve infant and maternal health are urgently needed.

## Notes

Supplement 1. **eTable 1.** Pregnancy-Specific Policy Descriptions **eTable 2.** Diagnosis Codes for Infant Injuries Associated With Maltreatment **eTable 3.** Diagnosis Codes for Infant Morbidities Associated With Alcohol Use During Pregnancy **eTable 4.** Diagnosis and Procedure Codes for Severe Maternal Morbidities **eTable 5.** Unadjusted Associations Between Pregnancy-Specific Alcohol Policies and Infant and Maternal Outcomes and Infant Health Care Utilization **eTable 6.** Number of Individuals in the Study Cohort Exposed to Each Policy in Each Estimated Year of Conception **eTable 7.** Relationships Between Pregnancy-Specific Alcohol Policies and Infant and Maternal Outcomes, Main Models Showing Odds Ratios for Individual-Level Controls and, When Included, State-Level Controls **eTable 8.** Relationships Between Pregnancy-Specific Alcohol Policies and Infant and Maternal Outcomes and Infant Health Care Utilization, With Policies in Effect When People Give Birth **eTable 9.** Relationships Between Pregnancy-Specific Drug Policies and Infant and Maternal Outcomes and Infant Health Care Utilization **eTable 10.** Relationships Between Pregnancy-Specific Alcohol and/or Drug Policies and Infant and Maternal Outcomes and Infant Health Care Utilization **eTable 11.** Relationships Between Pregnancy-Specific Alcohol Policies and Infant Healthcare Utilization, Main Models Showing Odds Ratios for Individual-Level Controls and, When Included, State-Level Controls

Supplement 2.
Data Sharing Statement

## References

[1] Broccia M, Munch A, Hansen BM, . Heavy prenatal alcohol exposure and overall morbidities: a Danish nationwide cohort study from 1996 to 2018. Lancet Public Health. 2023;8(1):e36-e46. doi:10.1016/S2468-2667(22)00289-4 36603909

[2] May PA, de Vries MM, Marais AS, . The prevalence of fetal alcohol spectrum disorders in rural communities in South Africa: a third regional sample of child characteristics and maternal risk factors. Alcohol Clin Exp Res. 2022;46(10):1819-1836. doi:10.1111/acer.14922 35971629 PMC9588757

[3] May PA, Chambers CD, Kalberg WO, . Prevalence of fetal alcohol spectrum disorders in 4 US communities. JAMA. 2018;319(5):474-482. doi:10.1001/jama.2017.21896 29411031 PMC5839298

[4] National Institute on Alcohol Abuse and Alcoholism. Alcohol Policy Information System. Accessed January 31, 2023. http://www.alcoholpolicy.niaaa.nih.gov/

[5] Centers for Disease Control and Prevention (CDC). Alcohol consumption among pregnant and childbearing-aged women—United States, 1991 and 1995. MMWR Morb Mortal Wkly Rep. 1997;46(16):346-350. 9148136

[6] Centers for Disease Control and Prevention (CDC). Alcohol use among women of childbearing age—United States, 1991-1999. MMWR Morb Mortal Wkly Rep. 2002;51(13):273-276. 11952279

[7] Centers for Disease Control and Prevention (CDC). Alcohol use among pregnant and nonpregnant women of childbearing age—United States, 1991-2005. MMWR Morb Mortal Wkly Rep. 2009;58(19):529-532. 19478721

[8] Zhao G, Ford ES, Tsai J, . Trends in health-related behavioral risk factors among pregnant women in the United States: 2001-2009. J Womens Health (Larchmt). 2012;21(3):255-263. doi:10.1089/jwh.2011.2931 22047097

[9] Denny CH, Acero CS, Naimi TS, Kim SY. Consumption of alcohol beverages and binge drinking among pregnant women aged 18-44 years—United States, 2015-2017. MMWR Morb Mortal Wkly Rep. 2019;68(16):365-368. doi:10.15585/mmwr.mm6816a1 31022164 PMC6483284

[10] Hasin DS, Shmulewitz D, Keyes K. Alcohol use and binge drinking among U.S. men, pregnant and non-pregnant women ages 18-44: 2002-2017. Drug Alcohol Depend. 2019;205:107590. doi:10.1016/j.drugalcdep.2019.107590 31600616 PMC6893082

[11] Roberts SCM, Mericle AA, Subbaraman MS, . State policies targeting alcohol use during pregnancy and alcohol use among pregnant women 1985-2016: evidence from the Behavioral Risk Factor Surveillance System. Womens Health Issues. 2019;29(3):213-221. doi:10.1016/j.whi.2019.02.001 30876695 PMC6597255

[12] Subbaraman MS, Roberts SCM. Costs associated with policies regarding alcohol use during pregnancy: results from 1972-2015 Vital Statistics. PLoS One. 2019;14(5):e0215670. doi:10.1371/journal.pone.0215670 31067248 PMC6505739

[13] Subbaraman MS, Thomas S, Treffers R, . Associations between state-level policies regarding alcohol use among pregnant women, adverse birth outcomes, and prenatal care utilization: results from 1972 to 2013 Vital Statistics. Alcohol Clin Exp Res. 2018;42(8):1511-1517. doi:10.1111/acer.13804 29912478 PMC6298847

[14] Syed S, Ashwick R, Schlosser M, Gonzalez-Izquierdo A, Li L, Gilbert R. Predictive value of indicators for identifying child maltreatment and intimate partner violence in coded electronic health records: a systematic review and meta-analysis. Arch Dis Child. 2021;106(1):44-53. doi:10.1136/archdischild-2020-319027 32788201 PMC7788194

[15] O’Leary CM, Elliott EJ, Nassar N, Bower C. Exploring the potential to use data linkage for investigating the relationship between birth defects and prenatal alcohol exposure. Birth Defects Res A Clin Mol Teratol. 2013;97(7):497-504. doi:10.1002/bdra.23142 23873815

[16] Chen J, Cox S, Kuklina EV, Ferre C, Barfield W, Li R. Assessment of incidence and factors associated with severe maternal morbidity after delivery discharge among women in the US. JAMA Netw Open. 2021;4(2):e2036148. doi:10.1001/jamanetworkopen.2020.36148 33528553 PMC7856547

[17] Centers for Disease Control and Prevention. How does CDC identify severe maternal morbidity? December 26, 2019. Accessed March 27, 2023. https://www.cdc.gov/reproductivehealth/maternalinfanthealth/smm/severe-morbidity-ICD.htm#print

[18] American Academy of Pediatrics. AAP schedule of well-child care visits. March 6, 2023. Accessed June 20, 2023. https://www.healthychildren.org/English/family-life/health-management/Pages/Well-Child-Care-A-Check-Up-for-Success.aspx

[19] California Department of Health Care Services. Well-child visits in the first 15 months of life. Updated March 21, 2021. Accessed June 20, 2023. https://www.dhcs.ca.gov/dataandstats/Pages/Well-childvisitsinthefirst15monthsoflife.aspx

[20] Moore BJ, White S, Washington R, Coenen N, Elixhauser A. Identifying increased risk of readmission and in-hospital mortality using hospital administrative data: the AHRQ Elixhauser Comorbidity Index. Med Care. 2017;55(7):698-705. doi:10.1097/MLR.0000000000000735 28498196

[21] Centers for Disease Control and Prevention. The tax burden on tobacco, 1970-2019. March 22, 2021. Accessed June 18, 2021. https://chronicdata.cdc.gov/Policy/The-Tax-Burden-on-Tobacco-1970-2019/7nwe-3aj9

[22] Nephew TM, Yi H, Williams GD, Stinson FS, Dufour MC. US alcohol epidemiologic data reference manual, Volume 1, 4th Edition. US apparent consumption of alcoholic beverages based on state sales, taxation, or receipt data. NIH Publication No. 04-5563. June 2004. Accessed June 29, 2023. https://pubs.niaaa.nih.gov/publications/DRM_04-5563/DRM_04-5563.pdf

[23] Slater ME, Alpert HR; National Institute on Alcohol Abuse and Alcoholism. Surveillance report #117: apparent per capita alcohol consumption—national, state, and regional trends, 1977-2019. April 2021. Accessed June 29, 2023. https://pubs.niaaa.nih.gov/publications/surveillance117/SR-117-Per-Capita-Consumption.pdf

[24] US Bureau of Labor Statistics. Employment status of the civilian noninstitutional population, annual averages. Accessed December 1, 2022. https://www.bls.gov/lau/rdscnp16.htm

[25] US Census Bureau. Table 19: number of poor and poverty rate by state. Accessed December 11, 2022. https://www.census.gov/data/tables/time-series/demo/income-poverty/historical-poverty-people.html

[26] Thomas S, Treffers R, Berglas NF, Drabble L, Roberts SCM. Drug use during pregnancy policies in the United States from 1970-2016. Contemp Drug Probl. 2018;45(4):441-459. doi:10.1177/0091450918790790 PMC12811024 41551655

[27] Faherty LJ, Kranz AM, Russell-Fritch J, Patrick SW, Cantor J, Stein BD. Association of punitive and reporting state policies related to substance use in pregnancy with rates of Neonatal Abstinence Syndrome. JAMA Netw Open. 2019;2(11):e1914078. doi:10.1001/jamanetworkopen.2019.14078 31722022 PMC6902764

[28] Meinhofer A, Witman A, Maclean JC, Bao Y. Prenatal substance use policies and newborn health. Health Econ. 2022;31(7):1452-1467. doi:10.1002/hec.4518 35445500 PMC9177792

[29] Woodruff K, Roberts SCM. “Alcohol during pregnancy? Nobody does that anymore”: state legislators’ use of evidence in making policy on alcohol use in pregnancy. J Stud Alcohol Drugs. 2019;80(3):380-388. doi:10.15288/jsad.2019.80.380 31250804 PMC6614924

[30] Andrews JG, Galindo MK, Meaney FJ, . Recognition of clinical characteristics for population-based surveillance of fetal alcohol syndrome. Birth Defects Res. 2018;110(10):851-862. doi:10.1002/bdr2.1203 29368410 PMC6066339

[31] O’Leary LA, Ortiz L, Montgomery A, ; FASSNetII. Methods for surveillance of fetal alcohol syndrome: the Fetal Alcohol Syndrome Surveillance Network II (FASSNetII)—Arizona, Colorado, New York, 2009-2014. Birth Defects Res A Clin Mol Teratol. 2015;103(3):196-202. doi:10.1002/bdra.23335 25761572 PMC4484746

[32] Putnam-Hornstein E, Needell B, Rhodes AE. Understanding risk and protective factors for child maltreatment: the value of integrated, population-based data. Child Abuse Negl. 2013;37(2-3):116-119. doi:10.1016/j.chiabu.2012.08.006 23260115

[33] Maclean JC, Witman A, Durrance CP, Atkins DN, Meinhofer A. Prenatal substance use policies and infant maltreatment reports. Health Aff (Millwood). 2022;41(5):703-712. doi:10.1377/hlthaff.2021.01755 35500191 PMC10035583

