# Error in Figure 1

**DOI:** 10.1001/jamanetworkopen.2023.40368

**Published:** 2023-10-13

In the Original Investigation titled “Association of Pregnancy-Specific Alcohol Policies With Infant Morbidities and Maltreatment,”^1^ published August 3, 2023, there was an error in Figure 1. The second-to-last box in the flowchart should have read, “1 575 197 Birthing persons aged ≥25 y” instead of “1 575 197 Birthing persons aged >25 y” to account for the inclusion of persons aged 25 years in the cohort. This article has been corrected.^1^
