# Supplementary material for: Association of Pregnancy-Specific Alcohol Policies With Infant Morbidities and Maltreatment
Source: JAMA Netw Open. 2023 Aug 3;6(8):e2327138. doi: 10.1001/jamanetworkopen.2023.27138 (PMC10401306; doi:10.1001/jamanetworkopen.2023.27138)
Supplement: Supplement 1. — eTable 1. Pregnancy-Specific Policy Descriptions eTable 2. Diagnosis Codes for Infant Injuries Associated With Maltreatment eTable 3. Diagnosis Codes for Infant Morbidities Associated With Alcohol Use During Pregnancy eTable 4. Diagnosis and Procedure Codes for Severe Maternal Morbidities eTable 5. Unadjusted Associations Between Pregnancy-Specific Alcohol Policies and Infant and Maternal Outcomes and Infant Health Care Utilization eTable 6. Number of Individuals in the Study Cohort Exposed to Each Policy in Each Estimated Year of Conception eTable 7. Relationships Between Pregnancy-Specific Alcohol Policies and Infant and Maternal Outcomes, Main Models Showing Odds Ratios for Individual-Level Controls and, When Included, State-Level Controls eTable 8. Relationships Between Pregnancy-Specific Alcohol Policies and Infant and Maternal Outcomes and Infant Health Care Utilization, With Policies in Effect When People Give Birth eTable 9. Relationships Between Pregnancy-Specific Drug Policies and Infant and Maternal Outcomes and Infant Health Care Utilization eTable 10. Relationships Between Pregnancy-Specific Alcohol and/or Drug Policies and Infant and Maternal Outcomes and Infant Health Care Utilization eTable 11. Relationships Between Pregnancy-Specific Alcohol Policies and Infant Healthcare Utilization, Main Models Showing Odds Ratios for Individual-Level Controls and, When Included, State-Level Controls [file jamanetwopen-e2327138-s001.pdf]

Supplemental Online Content

Roberts SCM, Schulte A, Zaugg C, Leslie DL, Corr TE, Liu G. Association of pregnancy-specific alcohol policies with infant morbidities and maltreatment. *JAMA Netw Open*. 2023;6(8):e2327138. doi:10.1001/jamanetworkopen.2023.27138

eTable 1. Pregnancy-Specific Policy Descriptions

eTable 2. Diagnosis Codes for Infant Injuries Associated With Maltreatment

eTable 3. Diagnosis Codes for Infant Morbidities Associated With Alcohol Use During Pregnancy

eTable 4. Diagnosis and Procedure Codes for Severe Maternal Morbidities

eTable 5. Unadjusted Associations Between Pregnancy-Specific Alcohol Policies and Infant and Maternal Outcomes and Infant Health Care Utilization

eTable 6. Number of Individuals in the Study Cohort Exposed to Each Policy in Each Estimated Year of Conception

eTable 7. Relationships Between Pregnancy-Specific Alcohol Policies and Infant and Maternal Outcomes, Main Models Showing Odds Ratios for Individual-Level Controls and, When Included, State-Level Controls

eTable 8. Relationships Between Pregnancy-Specific Alcohol Policies and Infant and Maternal Outcomes and Infant Health Care Utilization, With Policies in Effect When People Give Birth

eTable 9. Relationships Between Pregnancy-Specific Drug Policies and Infant and Maternal Outcomes and Infant Health Care Utilization

eTable 10. Relationships Between Pregnancy-Specific Alcohol and/or Drug Policies and Infant and Maternal Outcomes and Infant Health Care Utilization

eTable 11. Relationships Between Pregnancy-Specific Alcohol Policies and Infant Healthcare Utilization, Main Models Showing Odds Ratios for Individual-Level Controls and, When Included, State-Level Controls

This supplemental material has been provided by the authors to give readers additional information about their work.

© 2023 Roberts SCM et al. *JAMA Network Open*.

eTable 1 Pregnancy-Specific Policy Descriptions	
Reporting requirements	Require reporting suspicion of or evidence of alcohol use or “abuse” by women during pregnancy to Child Protective Services (CPS) or health authority. Reporting may be for 1) child welfare purposes (child abuse/neglect investigation), 2) referral for assessment and/or treatment, or 3) data gathering purposes.
Mandatory warning signs	Require that notices warning of harm from consumption during pregnancy be posted in licensed settings, where alcohol is sold.
Child abuse/child neglect	Address legal significance of woman’s conduct prior to birth of a child and of damage caused in utero and, in some cases, define alcohol use during pregnancy as child abuse or neglect.
Civil commitment	Mandatory involuntary commitment of a pregnant woman to treatment or mandatory involuntary placement of a pregnant woman in protective custody of the state for the protection of a fetus from prenatal exposure to alcohol.
Prohibitions against criminal prosecution	Prohibit use of medical test results, such as prenatal screenings or toxicology tests, as evidence in criminal prosecutions of women who may have caused harm to a fetus or a child.
Priority treatment	Mandate priority access to substance use disorder treatment for 1) pregnant women who “abuse alcohol” and 2) pregnant women who “abuse alcohol” and women with children.

© 2023 Roberts SCM et al. *JAMA Network Open*.

eTable 2. Diagnosis Codes for Infant Injuries Associated With Maltreatment		
**Diagnosis name**	**ICD9 diagnosis codes**	**ICD10 diagnosis codes**
child maltreatment primary diagnosis (cm)	995.5, E96.0-E96.8	T74, Y06, Y07
abusive head trauma	cm + 800, 801, 781.0-781.4, 854.0, 950.0-950.3	cm + S06, S09.7, S09.8, T90.5
assaults	E96.x, V71.6	X85-Y09
assaults/child maltreatment/adversity	V15.4, V61.2, V71.8, E96.7, E96.01, & cm	Y04.0X, Y04.2X, Y04.8X, Y07.41-Y07.43, Y07.49, Y07.50-Y07.53, Y07.59, Y07.0-Y07.1, Y07.9, Z03.6, Z03.8, Z04.7, Z62.0-Z62.1, Z62.3, Z62.6, Z62.9, Z62.81-Z62.82, Z62.89, Z69.01-Z69.02, Z91.41, Z91.49, & cm
retinal haemorrhage	362.81	H35.6
rib fractures	807.0- 807.1	S22.3- S22.4
subdural hematoma	852.2, 432.1	I62.0, S06.57
These include diagnoses with a positive predictive value >50% with maltreatment, based on this systematic review. Syed S, Ashwick R, Schlosser M, Gonzalez-Izquierdo A, Li L, Gilbert R. Predictive value of indicators for identifying child maltreatment and intimate partner violence in coded electronic health records: a systematic review and meta-analysis. *Arch Dis Child.* 2021;106(1):44-53.		

© 2023 Roberts SCM et al. *JAMA Network Open*.

eTable 3. Diagnosis Codes for Infant Morbidities Associated With Alcohol Use During Pregnancy		
**Diagnosis name**	**ICD9 diagnosis codes**	**ICD10 diagnosis codes**
ventricular and atrioventricular septal defects	745.4, 745.6	Q21.0, Q21.2
discordant ventriculoarterial connection; discordant atrioventricular connection; other congenital malformations of the cardiac chambers and connections, unspecified	745.1	Q20.3, Q20.5, Q20.8
common arterial trunk, persistent truncus arteriosus	745.0	Q20.0
interruption of aortic arch; other congenital malformations of aorta	747.11, 747.10	Q25.1
double outlet right ventricle; double outlet left ventricle	745.11, 745.19	Q20.1, Q20.2
tetralogy of fallot	754.2	Q21.3
lobulated, fused and horseshoe kidney	753.3	Q63.1
duplication of ureter	753.4	Q62.5
renal dysplasia, renal hypoplasia, unilateral/bilateral/unspecified	753.0, 753.15	Q61.4, Q60.3, Q60.4, Q60.5
congenital hydronephrosis	753.2x (x=0, 1, 2, 3, 9)	Q62.0
congenital ptosis	743.61	Q10.0
optic nerve hypoplasia, unspecified eye	377.43	H47.039
other congenital malformations of spine, not associated with scoliosis	756.1x (x=0-7, 9)	Q76.4
congenital deformity of spine; congenital scoliosis due to congenital bony malformation	754.2	Q67.5, Q76.3
other congenital malformations of nails	757.5	Q84.6
congenital absence of unspecified hand and finger	755.39	Q71.30
other congenital malformations of upper limb(s), including shoulder girdle	755.53, 755.59	Q74.0
contracture of joint	718.4x	M24.5x (x=0-7, 9)
pectus excavatum, pectus carinatum	754.81, 754.82	Q67.6, Q67.7
microcephaly	742.1	Q02
congenital anomalies of the nervous system	740x (x=0-2), 742x (x=0-5, 8, 9; 5 can have 5th digit of 1, 3, 9)	Q00x-Q07x
FAS (dysmorphism); newborn affected by maternal use of alcohol	760.71	Q86.0, P04.3,
congenital malformation of face and neck, unspecified	744.9	Q18.9

© 2023 Roberts SCM et al. *JAMA Network Open*.

eTable 4. Diagnosis and Procedure Codes for Severe Maternal Morbidities

**Diagnosis name**	**ICD9 diagnosis codes**	**ICD10 diagnosis codes**
acute myocardial infarction	410.xx	I21.xx, I22.xx
aneurysm	441.xx	I71.xx, I79.0
acute renal failure	584.5-584.9, 669.3x	N17.x, O90.4
adult respiratory distress syndrome	518.5x, 518.81-518.82, 518.84, 799.1	J80, J95.1- J95.3, J95.82x, J96.0, J96.2, R092
amniotic fluid embolism	673.1x	O881.x
cardiac arrest/ventricular fibrillation	427.41- 427.42, 427.5	I46x, I49.0x
conversion of cardiac rhythm	99.6x	5A12012, 5A2204Z
disseminated intravascular coagulation	286.6, 286.9, 666.3x	D65; D688, D689, O723
eclampsia	642.6x	O15x
heart failure/arrest during surgery or procedure	669.4x, 997.1	I97.12x-I97.13x, I97.710-I97.711
puerperal cerebrovascular disorders	430.xx-434.xx, 436.xx- 437.xx, 671.5x, 674.0x, 997.02	I60.xx-I68.xx, I62.9, I97.81x-I97.82x, O22.51-O22.53, O87.3
pulmonary edema and acute heart failure	428.0, 428.1, 428.21, 428.23, 428.31, 428.33, 428.41, 428.43, 518.4	I50.1, I50.9, I50.20-I50.21, I50.23, I50.30-I50.31, I50.33, I504.0-I50.41, I50.43, J81.0
severe anesthesia complications	668.0x, 668.1x, 668.2x	O74.0-O74.3, O89.0x-O89.2
sepsis	038.xx, 670.2x, 995.91-995.92	A32.7, A40.x, A41.x, O85, O86.04, R65.20, T81.44xx, T80.211A, T81.4XXA
shock	669.1x, 785.5x, 995.0, 995.4, 998.0x	O75.1, R57.x, R65.21, T78.2XXA, T88.2XXA, T88.6XXA, T81.10XA, T81.11XA, T81.19XA
sickle cell disease with crisis	282.42, 282.62, 282.64, 282.69	D57.0x, D57.21x, D57.41x, D57.81x
air and thrombotic embolism	415.1x, 673.0x, 673.2x, 673.3x, 673.8x	I26.x, O88.0x, O88.2x, O88.3x, O88.8x
blood products transfusion	99.0x	First 5=30233, 30230, 30240, 30243, 30250, 30253, 30260, 30263; Last 2=Hx, Kx, Lx, Mx, Nx, Px, Rx, Tx with x as 0 or 1
hysterectomy	68.3x-68.9x	0UT90ZZ, 0UT94ZZ, 0UT97ZZ, 0UT98ZZ, 0UT9FZZ
temporary tracheostomy	31.1	0B110Z, 0B110F, 0B113, 0B114
ventilation	93.90, 96.01-96.03, 96.05	5A1935Z, 5A1945Z, 5A1955Z

© 2023 Roberts SCM et al. *JAMA Network Open*.

eTable 5. Unadjusted Associations Between Pregnancy-Specific Alcohol Policies and Infant and Maternal Outcomes and Infant Health Care Utilization			
	Infant injuries associated with maltreatment	Infant morbidities associated with alcohol use during pregnancy	Severe maternal morbidities
	OR (95% CI)	OR (95% CI)	OR (95% CI)
Reporting requirements CPS	1.01(0.80-1.26)	1.00(0.90-1.11)	1.10(0.70-1.73)
Reporting requirements data	0.97(0.77-1.23)	1.06(0.97-1.15)	0.98(0.61-1.58)
Reporting requirements assessment/treatment	1.17(1.00-1.37)	1.04(0.99-1.09)	1.26(0.85-1.87)
Mandatory Warning Signs	1.23(1.14-1.33)	1.03(0.93-1.14)	1.83(1.68-1.99)
Child abuse/neglect	0.92(0.72-1.16)	1.05(1.00-1.11)	0.88(0.36-2.11)
Civil Commitment	1.40(1.26-1.55)	0.56(0.54-0.58)	1.14(0.96-1.34)
Limits on Criminal Prosecution	1.09(1.02-1.18)	1.11(0.97-1.26)	1.00(0.62-1.61)
Priority Treatment Pregnant women only	0.81(0.75-0.88)	0.96(0.88-1.06)	0.96(0.88-1.04)
Priority Treatment Pregnant women & women with children	1.15(1.03-1.28)	1.07(1.03-1.12)	0.80(0.67-0.97)
	Inadequate well-child visits	2+ Emergency Department visits	2+ Inpatient admissions
	OR (95% CI)	OR (95% CI)	OR (95% CI)
Reporting requirements CPS	0.85(0.71-1.00)	1.09(1.00-1.18)	1.09(0.85-1.39)
Reporting requirements data	0.93(0.80-1.08)	1.08(0.96-1.23)	0.97(0.82-1.15)
Reporting requirements assessment/treatment	0.90(0.80-1.01)	1.07(0.99-1.14)	0.88(0.76-1.02)
Mandatory Warning Signs	1.09(0.99-1.2)	1.05(0.99-1.11)	0.87(0.81-0.93)
Child abuse/neglect	0.94(0.79-1.13)	1.06(0.90-1.26)	0.98(0.80-1.2)
Civil Commitment	0.95(0.81-1.1)	1.24(1.16-1.32)	1.17(1.12-1.22)
Limits on Criminal Prosecution	0.85(0.73-1.00)	0.88(0.78-0.99)	0.94(0.80-1.10)
Priority Treatment Pregnant women only	1.10(1.01-1.20)	0.99(0.95-1.03)	0.94(0.91-0.98)
Priority Treatment Pregnant women & women with children	1.07(0.91-1.25)	0.95(0.89-1.02)	0.89(0.85-0.94)
These unadjusted models include each policy in a separate model, along with individual-level controls, state & year fixed effects, and state-specific time trends. They do not include state-controls, and do not adjust for the other pregnancy-specific policies.			

© 2023 Roberts SCM et al. *JAMA Network Open*.

eTable 6. Number of Individuals in the Study Cohort Exposed to Each Policy in Each Estimated Year of Conception

	Total	2005	2006	2007	2008	2009	2010	2011	2012	2013	2014	2015	2016	2017	2018	2019
Reporting requirements CPS	603,578	21,093	31,221	37,835	41,461	41,102	55,135	63,268	56,780	44,491	47,728	43,603	37,012	38,102	38,065	6,682
Reporting requirements data	848,615	27,187	56,227	65,280	67,849	65,067	87,533	79,767	72,878	57,847	54,746	50,580	46,502	56,101	51,939	9,112
Reporting requirements assessment/treatment	638,965	16,271	31,004	38,153	48,030	49,912	65,999	67,454	59,461	48,203	48,925	46,596	36,996	39,507	35,923	6,531
Mandatory Warning Signs	773,430	24,533	42,638	52,490	66,767	65,961	84,744	75,524	68,910	58,754	54,153	49,500	39,790	43,135	39,547	6,984
Child abuse/neglect	652,769	22,008	48,313	54,921	52,937	48,630	63,615	52,859	49,910	46,413	50,288	46,222	36,284	36,956	36,725	6,683
Civil Commitment	61,633	1,357	2,899	4,609	6,003	5,574	6,493	5,311	5,332	4,498	4,590	3,529	2,557	3,992	4,224	665
Limits on Criminal Prosecution	135,968	5,952	8,307	9,104	8,474	7,849	10,465	10,775	11,782	11,672	13,188	12,120	8,088	8,690	8,084	1,418
Priority Treatment Pregnant women only	362,879	16,854	25,551	27,034	25,586	24,472	34,044	34,305	34,612	34,528	31,937	24,281	15,443	16,919	14,766	2,547
Priority Treatment Pregnant women & women with children	162,584	6,950	16,967	18,178	20,222	18,422	23,038	10,687	8,750	7,929	7,687	6,620	5,917	5,522	4,832	863

© 2023 Roberts SCM et al. *JAMA Network Open*.

eTable 7. Relationships Between Pregnancy-Specific Alcohol Policies and Infant and Maternal Outcomes, Main Models Showing Odds Ratios for Individual-Level Controls and, When Included, State-Level Controls			
	Infant injuries associated with maltreatment	Infant morbidities associated with alcohol use during pregnancy	Severe maternal morbidities
	aOR (95% CI)	aOR (95% CI)	aOR (95% CI)
Reporting requirements CPS	1.00 (0.76-1.31)	0.96 (0.79-1.18)	1.19 (0.73-1.94)
Reporting requirements data	0.94 (0.81-1.09)	1.05 (0.96-1.15)	0.95 (0.56-1.60)
Reporting requirements assessment/treatment	1.28 (1.08-1.52)	1.04 (0.94-1.16)	1.35 (0.92-2.00)
Mandatory Warning Signs	1.18 (1.10-1.27)	1.01 (0.93-1.11)	1.87 (1.72-2.03)
Child abuse/neglect	0.82 (0.63-1.07)	1.02 (0.89-1.17)	0.69 (0.30-1.62)
Civil Commitment	1.26 (1.08-1.48)	0.57 (0.53-0.62)	1.10 (0.87-1.38)
Limits on Criminal Prosecution	1.12 (0.98-1.29)	1.10 (0.96-1.26)	1.05 (0.75-1.48)
Priority Treatment Pregnant women only	0.83 (0.76-0.90)	0.97 (0.89-1.05)	1.07 (0.99-1.16)
Priority Treatment Pregnant women & women with children	1.12 (1.00-1.25)	1.08 (1.03-1.13)	0.83 (0.70-0.97)
Age 25-29	1.04 (1.01-1.07)	0.96 (0.93-0.98)	0.95 (0.91-0.99)
Age 30-35	ref	ref	ref
Age 36-39	1.04 (1.00-1.08)	1.09 (1.07-1.12)	1.15 (1.12-1.18)
Age 40-44	1.17 (1.10-1.24)	1.31 (1.26-1.36)	1.34 (1.29-1.39)
Age 45+	1.58 (1.34-1.86)	1.57 (1.37-1.81)	1.87 (1.65-2.11)
0 Elixhauser comorbidities	ref	ref	ref
1 Elixhauser comorbidity	1.19 (1.15-1.22)	1.17 (1.13-1.20)	1.33 (1.29-1.38)
2 Elixhauser comorbidities	1.39 (1.34-1.45)	1.34 (1.29-1.40)	1.74 (1.64-1.83)
3 + Elixhauser comorbidities	1.69 (1.60-1.79)	1.55 (1.46-1.64)	2.67 (2.47-2.89)
Unemployment	1.04 (0.98-1.11)	--	--
Poverty	--	--	--
Per capita tobacco consumption	--	--	--
Models also include state and year fixed effects, state-specific time trends, and account for clustering by state.			

© 2023 Roberts SCM et al. *JAMA Network Open*.

eTable 8. Relationships Between Pregnancy-Specific Alcohol Policies and Infant and Maternal Outcomes and Infant Health Care Utilization, With Policies in Effect When People Give Birth

	Infant injuries associated with maltreatment	Infant morbidities associated with alcohol use during pregnancy	Severe maternal morbidities
	aOR (95% CI)	aOR (95% CI)	aOR (95% CI)
Reporting requirements CPS	1.03 (0.88-1.21)	0.89 (0.72-1.10)	1.50 (1.03-2.18)
Reporting requirements data	1.06 (0.91-1.24)	1.17 (1.08-1.28)	1.06 (0.86-1.30)
Reporting requirements assessment/treatment	1.27 (1.08-1.49)	1.01 (0.90-1.14)	1.13 (0.78-1.63)
Mandatory Warning Signs	1.04 (0.95-1.15)	0.98 (0.94-1.02)	1.42 (1.33-1.52)
Child abuse/neglect	0.97 (0.84-1.13)	1.16 (0.98-1.39)	0.71 (0.40-1.25)
Civil Commitment	1.04 (0.92-1.17)	0.87 (0.79-0.96)	1.16 (0.96-1.40)
Limits on Criminal Prosecution	0.96 (0.84-1.10)	0.94 (0.88-1.01)	1.00 (0.64-1.56)
Priority Treatment Pregnant women only	0.76 (0.71-0.82)	0.93 (0.89-0.96)	0.85 (0.70-1.03)
Priority Treatment Pregnant women & women with children	1.21 (1.05-1.40)	1.17 (1.12-1.23)	0.95 (0.80-1.12)
	Inadequate well-child visits	2+ Emergency Department visits	2+ Inpatient admissions
	aOR (95% CI)	aOR (95% CI)	aOR (95% CI)
Reporting requirements CPS	0.70 (0.53-0.92)	1.03 (0.91-1.18)	1.20 (1.00-1.43)
Reporting requirements data	1.05 (0.90-1.23)	1.11 (1.04-1.18)	1.12 (1.01-1.25)
Reporting requirements assessment/treatment	1.09 (0.81-1.46)	0.98 (0.84-1.16)	0.78 (0.66-0.91)
Mandatory Warning Signs	1.35 (1.24-1.46)	0.88 (0.83-0.93)	1.04 (0.97-1.12)
Child abuse/neglect	1.25 (1.05-1.47)	1.06 (0.94-1.19)	0.86 (0.70-1.05)
Civil Commitment	1.09 (0.93-1.28)	1.07 (1.00-1.15)	0.81 (0.72-0.92)
Limits on Criminal Prosecution	0.84 (0.75-0.95)	0.70 (0.66-0.75)	1.08 (0.91-1.29)
Priority Treatment Pregnant women only	1.04 (0.95-1.14)	1.00 (0.96-1.03)	1.03 (0.96-1.09)
Priority Treatment Pregnant women & women with children	1.12 (0.86-1.48)	0.96 (0.91-1.01)	1.02 (0.93-1.12)

Models include individual-level controls (age, health status), state and year fixed effects, state-specific time trends, and account for clustering by state. The infant injuries model also controls for state-level unemployment. The emergency department visits model also controls for state-level unemployment and poverty; the inpatient admissions model also controls for state-level per capita tobacco consumption.

© 2023 Roberts SCM et al. *JAMA Network Open*.

eTable 9. Relationships Between Pregnancy-Specific Drug Policies and Infant and Maternal Outcomes and Infant Health Care Utilization			
	Infant injuries associated with maltreatment	Infant morbidities associated with alcohol use during pregnancy	Severe maternal morbidities
	aOR (95% CI)	aOR (95% CI)	aOR (95% CI)
Reporting requirements CPS	1.04 (0.79-1.37)	0.98 (0.79-1.20)	0.82 (0.67-1.00)
Reporting requirements data	0.89 (0.73-1.07)	1.00 (0.92-1.09)	0.93 (0.68-1.27)
Reporting requirements assessment/treatment	1.11 (0.94-1.31)	1.09 (0.93-1.27)	1.16 (0.89-1.51)
Mandatory Warning Signs	0.78 (0.67-0.91)	1.05 (0.87-1.27)	1.05 (0.71-1.54)
Child abuse/neglect	0.89 (0.63-1.28)	0.99 (0.85-1.15)	1.46 (0.97-2.21)
Civil Commitment	1.34 (1.21-1.49)	0.56 (0.54-0.59)	1.13 (0.96-1.34)
Limits on Criminal Prosecution	0.98 (0.74-1.30)	1.04 (0.88-1.22)	1.04 (0.67-1.62)
Priority Treatment Pregnant women only	0.87 (0.79-0.96)	0.96 (0.88-1.04)	1.04 (0.88-1.23)
Priority Treatment Pregnant women & women with children	1.09 (0.72-1.64)	1.02 (0.91-1.14)	0.92 (0.80-1.05)
	Inadequate well-child visits	2+ Emergency Department visits	2+ Inpatient admissions
	aOR (95% CI)	aOR (95% CI)	aOR (95% CI)
Reporting requirements CPS	0.80 (0.65-0.99)	1.04 (0.97-1.12)	1.23 (0.95-1.60)
Reporting requirements data	0.94 (0.80-1.11)	1.03 (0.94-1.13)	1.02 (0.90-1.14)
Reporting requirements assessment/treatment	1.01 (0.83-1.23)	1.03 (0.96-1.10)	0.88 (0.76-1.02)
Mandatory Warning Signs	0.63 (0.42-0.94)	0.95 (0.85-1.07)	0.96 (0.85-1.10)
Child abuse/neglect	1.18 (0.95-1.46)	1.10 (0.99-1.22)	0.92 (0.71-1.19)
Civil Commitment	0.93 (0.79-1.10)	1.28 (1.22-1.35)	1.17 (1.12-1.23)
Limits on Criminal Prosecution	0.96 (0.79-1.18)	0.85 (0.78-0.92)	0.89 (0.81-0.98)
Priority Treatment Pregnant women only	1.06 (0.99-1.13)	0.99 (0.95-1.03)	0.95 (0.91-0.99)
Priority Treatment Pregnant women & women with children	1.12 (0.98-1.27)	0.91 (0.85-0.98)	1.03 (0.98-1.08)
Models include individual-level controls (age, health status), state and year fixed effects, state-specific time trends, and account for clustering by state. The infant injuries model also controls for state-level unemployment. The emergency department visits model also controls for state-level unemployment and poverty; the inpatient admissions model also controls for state-level per capita tobacco consumption.			

© 2023 Roberts SCM et al. *JAMA Network Open*.

Table 10. Relationships Between Pregnancy-Specific Alcohol and/or Drug Policies and Infant and Maternal Outcomes and Infant Health Care Utilization			
	Infant injuries associated with maltreatment	Infant morbidities associated with alcohol use during pregnancy	Severe maternal morbidities
	aOR (95% CI)	aOR (95% CI)	aOR (95% CI)
Reporting requirements CPS	1.10 (0.83-1.44)	0.99 (0.80-1.23)	0.82 (0.67-1.01)
Reporting requirements data	0.90 (0.78-1.03)	1.01 (0.92-1.10)	0.99 (0.69-1.44)
Reporting requirements assessment/treatment	1.24 (1.05-1.47)	1.06 (0.94-1.19)	1.14 (0.90-1.44)
Mandatory Warning Signs	1.21 (1.10-1.33)	1.01 (0.92-1.10)	1.91 (1.72-2.12)
Child abuse/neglect	0.73 (0.52-1.02)	1.01 (0.83-1.22)	1.54 (0.87-2.72)
Civil Commitment	1.36 (1.22-1.51)	0.56 (0.54-0.59)	1.18 (1.05-1.33)
Limits on Criminal Prosecution	0.99 (0.75-1.32)	1.02 (0.87-1.20)	1.04 (0.67-1.61)
Priority Treatment Pregnant women only	0.89 (0.81-0.99)	0.96 (0.89-1.03)	1.14 (0.98-1.33)
Priority Treatment Pregnant women & women with children	0.95 (0.68-1.33)	1.02 (0.91-1.15)	0.83 (0.73-0.94)
	Inadequate well-child visits	2+ Emergency Department visits	2+ Inpatient admissions
	aOR (95% CI)	aOR (95% CI)	aOR (95% CI)
Reporting requirements CPS	0.81 (0.67-0.97)	1.05 (0.97-1.13)	1.30 (1.00-1.70)
Reporting requirements data	1.01 (0.86-1.18)	1.02 (0.89-1.17)	1.04 (0.93-1.17)
Reporting requirements assessment/treatment	0.97 (0.83-1.13)	1.02 (0.94-1.10)	0.83 (0.73-0.95)
Mandatory Warning Signs	1.11 (1.00-1.23)	1.03 (0.96-1.11)	0.85 (0.79-0.92)
Child abuse/neglect	1.24 (0.95-1.63)	1.11 (0.97-1.28)	0.84 (0.63-1.11)
Civil Commitment	0.95 (0.80-1.12)	1.28 (1.22-1.35)	1.16 (1.11-1.21)
Limits on Criminal Prosecution	0.94 (0.75-1.16)	0.84 (0.77-0.92)	0.88 (0.81-0.96)
Priority Treatment Pregnant women only	1.07 (1.00-1.15)	0.99 (0.95-1.03)	0.93 (0.89-0.97)
Priority Treatment Pregnant women & women with children	1.08 (0.97-1.20)	0.95 (0.88-1.03)	0.90 (0.83-0.98)

Models include individual-level controls (age, health status), state and year fixed effects, state-specific time trends, and account for clustering by state. The infant injuries model also controls for state-level unemployment. The emergency department visits model also controls for state-level unemployment and poverty; the inpatient admissions model also controls for state-level per capita tobacco consumption.

© 2023 Roberts SCM et al. *JAMA Network Open*.

eTable 11. Relationships Between Pregnancy-Specific Alcohol Policies and Infant Healthcare Utlization, Main Models Showing Odds Ratios for Individual-Level Controls and, When Included, State-Level Controls

	Inadequate well-child visits	2+ Emergency Department visits	2+ Inpatient admissions
	aOR (95% CI)	aOR (95% CI)	aOR (95% CI)
Reporting requirements CPS	0.83 (0.66-1.03)	1.09 (0.99-1.20)	1.24 (0.92-1.68)
Reporting requirements data	0.94 (0.80-1.11)	1.09 (0.98-1.22)	1.01 (0.89-1.15)
Reporting requirements assessment/treatment	0.97 (0.83-1.14)	0.99 (0.92-1.07)	0.81 (0.69-0.95)
Mandatory Warning Signs	1.12 (1.01-1.24)	1.03 (0.96-1.11)	0.84 (0.79-0.90)
Child abuse/neglect	1.12 (0.86-1.45)	1.04 (0.88-1.23)	0.94 (0.73-1.20)
Civil Commitment	0.98 (0.80-1.21)	1.31 (1.21-1.43)	1.12 (0.98-1.27)
Limits on Criminal Prosecution	0.91 (0.79-1.06)	0.82 (0.77-0.87)	0.88 (0.82-0.94)
Priority Treatment Pregnant women only	1.13 (1.03-1.23)	0.99 (0.95-1.03)	0.91 (0.88-0.95)
Priority Treatment Pregnant women & women with children	1.06 (0.90-1.24)	0.99 (0.93-1.04)	0.87 (0.83-0.92)
Age 25-29	1.16 (1.13-1.19)	1.25 (1.22-1.29)	1.16 (1.12-1.20)
Age 30-35	ref	ref	ref
Age 36-39	1.04 (1.01-1.07)	0.94 (0.93-0.95)	0.95 (0.92-0.98)
Age 40-44	1.17 (1.12-1.23)	0.96 (0.93-1.00)	1.09 (1.03-1.16)
Age 45+	1.16 (1.05-1.29)	1.04 (0.92-1.18)	1.45 (1.23-1.71)
0 Elixhauser comorbidities	ref	ref	ref
1 Elixhauser comorbidity	1.04 (1.02-1.06)	1.40 (1.37-1.43)	1.32 (1.28-1.35)
2 Elixhauser comorbidities	1.18 (1.14-1.23)	1.85 (1.81-1.91)	1.68 (1.60-1.76)
3 + Elixhauser comorbidities	1.35 (1.29-1.42)	2.51 (2.39-2.63)	2.29 (2.16-2.43)
Unemployment	--	0.98 (0.96-1.01)	--
Poverty	--	0.99 (0.98-1.00)	--
Per capita tobacco consumption	--	--	1.00 (1.00-1.01)

Models also include state and year fixed effects, state-specific time trends, and account for clustering by state.

© 2023 Roberts SCM et al. *JAMA Network Open*.
