# Supplementary material for: Association of Pregnancy-Specific Alcohol Policies With Infant Morbidities and Maltreatment
Source: JAMA Netw Open. 2023 Aug 3;6(8):e2327138. doi: 10.1001/jamanetworkopen.2023.27138 (PMC10401306; doi:10.1001/jamanetworkopen.2023.27138)
Supplement: Supplement 2. — Data Sharing Statement [file jamanetwopen-e2327138-s002.pdf]

Data Sharing Statement

Roberts. Association of Pregnancy-Specific Alcohol Policies With Infant Morbidities and Maltreatment. *JAMA Netw Open*. Published August 03, 2023.
doi:10.1001/jamanetworkopen.2023.27138

Data
Data available: No

Additional Information
Explanation for why data not available: Data on pregnancy-specific alcohol and drug policies are publicly available from the Alcohol Policy Information System. This study is a retrospective analysis of insurance claims data; primary data were not collected for the purposes of this study.
